# Repeatability of Motion Health Screening Scores Acquired from a Three-Dimensional Markerless Motion Capture System

**DOI:** 10.3390/jfmk7030065

**Published:** 2022-09-02

**Authors:** Dimitrije Cabarkapa, Damjana V. Cabarkapa, Nicolas M. Philipp, Gabriel G. Downey, Andrew C. Fry

**Affiliations:** Jayhawk Athletic Performance Laboratory—Wu Tsai Human Performance Alliance, Department of Health, Sport and Exercise Sciences, University of Kansas, Lawrence, KS 66045, USA

**Keywords:** biomechanics, measurement, technology, assessment, human motion, exercise

## Abstract

The purpose of the present study was to examine the repeatability of five algorithm-derived motion health screening scores (i.e., readiness, explosiveness, functionality, quality, and dysfunction) obtained from an innovative three-dimensional markerless motion capture system, composed of eight high-definition cameras recording at 60 fps. Thirteen females and six males performed two sets of three motion capture screenings, separated one week apart (six in total). The screenings consisted of 20 body movements performed in sequential order. Each screening within a testing session was separated by a 30 min rest interval to avoid the possible influence of fatigue. A trained research team member, facing the participant and standing outside of the camera capture range, was present to demonstrate each individual movement. The order in which motions were performed was identical across all participants. Repeated measures analysis of variance and intraclass correlation coefficients were used to examine statistically significant differences and measurement agreement across six testing sessions. The findings of the present study revealed no significant differences in algorithm-based motion health screening scores across multiple testing sessions. Moreover, excellent measurement reliability was found for readiness scores (ICC, 95% CI; 0.957, 0.914–0.980), good-to-excellent for functionality (0.905, 0.821–0.959) and explosiveness scores (0.906, 0.822–0.959), and moderate-to-excellent for dysfunction (0.829, 0.675–0.925) and quality scores (0.808, 0.635–0.915).

## 1. Introduction

Motion capture technology has experienced exponential growth over the last couple of decades. Both marker-based and markerless motion capture systems have been developed with hopes of providing health practitioners and sport scientists with an in-depth biomechanical analysis of various types of human movement patterns. The data obtained from these systems can be used to decrease the likelihood of injury, optimize and track recovery progress, and/or identify areas for further improvements in performance [1,2,3].

Currently, three-dimensional marker-based motion capture systems (e.g., Vicon, Oxford, UK) are considered to be a gold standard testing modality for examining human locomotion, primarily gait analysis and postural control [4,5,6,7,8]. Although being capable of providing precise and accurate measurements, their usage is predominantly limited to laboratory-based settings and is not without shortfalls [9]. A considerable amount of time is required to prepare the subject for testing procedures, which is not applicable in clinical and/or sport settings where data needs to be rapidly collected and analyzed [10]. Markers attached to the skin by double adhesive tape can influence normal movement patterns and can move relative to the underlying bone, commonly known as skin movement artifact [9]. Although most biomechanical variables of interest exhibited high repeatability, Ferber et al. [11] found that within-day testing comparisons were more reliable than between-day comparisons. The lower agreement seen in between-day repeatability scores may be attributed to the marker reapplication error, regardless of controlling for intertester variability [11]. In addition, it has been found that marker roundness, size, reflection capacity, as well as environmental conditions (e.g., lighting) are factors that need to be considered prior to data collection to minimize unnecessary measurement error [12].

A possible solution for some of the previously mentioned issues might lay in markerless motion capture technologies that are not prone to marker placement measurement error. An innovative single-based camera system (i.e., Microsoft Kinect, Microsoft Corp., Redmond, WA, USA) has demonstrated strong intertrial reliability and excellent concurrent validity when compared to a multicamera marker-based system (i.e., Vicon) for the majority of kinematic measurements performed during postural control tests, such as single-leg standing balance and the forward and lateral reach tests [8]. On the other hand, the same single-based camera system did not demonstrate as a feasible testing alternative for gait analysis in clinical settings, as it generally tended to underestimate hip and knee joint flexion and overestimate extension [4]. Moreover, despite being an inexpensive testing modality, this motion capture tracking modality has shown imprecision when measuring delicate motions such as ankle flexion/extension [13].

One of the possible solutions for improving the accuracy of markerless motion capture systems is to add additional cameras and use the visual hull construction process to track various types of human movement [9]. Harsted et al. [7] examined agreement between a markerless motion capture system composed of eight cameras (i.e., The Captury, GmbH, Saarbruken, Germany) and a marker-based system as a criterion measure (i.e., Vicon) for assessment of lower body kinematics and jump characteristics in pre-school children. While knee varus measurements were within unacceptable ranges, jump length, jump height, knee flexion, and knee–hip and ankle–hip separation distance measurements displayed acceptable levels of agreement and reliability [7]. In a recently published study, Cabarkapa et al. [14] tested the validity of a similar markerless motion capture system (i.e., DARI Motion, Overland Park, KS, USA) for the assessment of kinetic characteristics of the basketball dunking motion. When compared to a laboratory-based force plate as a criterion measure, peak force and peak power measurements demonstrated strong levels of agreement and consistency [14]. In addition, algorithm-derived functional movement screening scores obtained from the same motion capture system demonstrated a moderate-to-strong positive correlation with maximal aerobic capacity, and a moderate-to-strong negative correlation with body fat percentage [15]. The scores were founded on a screening protocol consisting of elementary body movements such as shoulder abduction/adduction and internal/external rotation, unilateral/bilateral squats, trunk rotation, and single-leg balance [15]. Overall, these findings are implying the further application of this type of motion capture technology in nonlaboratory settings with a diverse spectrum of the population (e.g., athletes, patients, and elderly individuals). 

While the usage and popularity of markerless three-dimensional motion capture technology is continuously growing due to its practical applicability in a clinical setting, it is of critical importance to establish confidence in measurements obtained from these systems. Therefore, the purpose of the present study was to examine the repeatability of five algorithm-derived motion health screening scores, obtained from an innovative three-dimensional markerless motion capture system across six testing sessions.

## 2. Materials and Methods

### 2.1. Participants

Thirteen females ($\bar{x}$ ± SD; age = 43.5 ± 13.4 years, height = 162.8 ± 5.8 cm, weight = 70.4 ± 19.1 kg, BMI = 26.5 ± 6.7 kg·m^−2^) and six males (age = 45.5 ± 10.2 years, height = 179.1 ± 7.4 cm, weight = 87.2 ± 10.2 kg, BMI = 27.2 ± 2.5 kg·m^−2^) volunteered to participate in this study. Participants with the following conditions were excluded from participation in the present study: (i) current orthopedic-related pain or injury, (ii) joint replacements (e.g., hip, knee, and/or shoulder), (iii) orthopedic-related surgery within the previous two years (including arthroscopic surgery for procedures such as ligament repair), (iv) currently under clinical care for serious health conditions (e.g., cancer, heart disease, stroke), and (v) previous participation in a three-dimensional motion screening assessment. All testing procedures performed in this study were approved by the Institutional Review Board. 

### 2.2. Procedures

All participants performed two sets of three motion capture screenings, separated one week apart, combining for a total of six screenings. The testing sessions were conducted at approximately the same time of the day and the participants were advised to maintain their regular exercise routine. Each screening within a testing session was separated by a 30 min rest interval to avoid a possible influence of fatigue. Prior to the initial testing session, participants’ anthropometric characteristics were assessed. Height was measured to the nearest tenth of a centimeter using a Harpenden stadiometer (Holtain Limited, London, UK). Body weight was measured to the nearest tenth of a kilogram using a Tanita scale (Tanita Corporation of America, IL, USA). Body mass index (BMI; kg·m^−2^) was calculated from the measured weight and height. 

**Figure 1:**
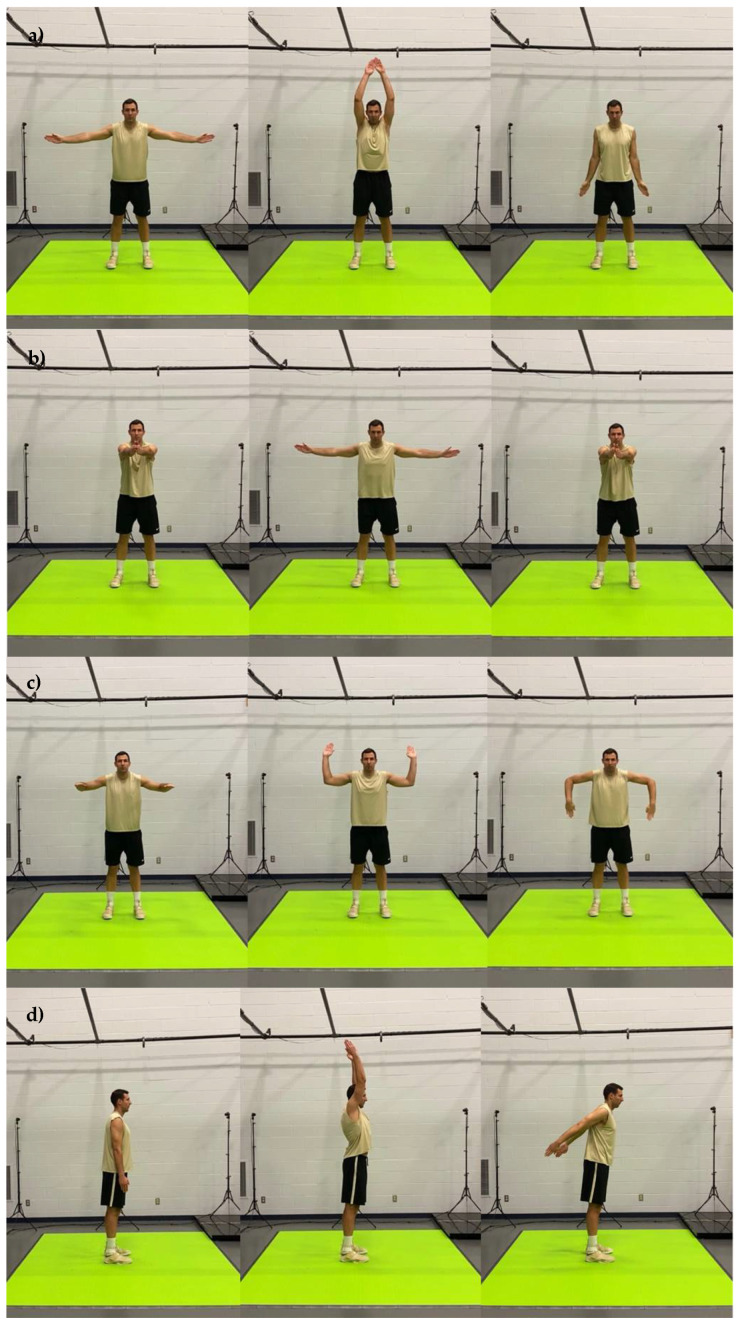
Graphical representation of body movements incorporated in motion health screening protocol; (**a**) shoulder abduction and adduction, (**b**) shoulder horizontal abduction, (**c**) shoulder internal and external rotation, (**d**) shoulder flexion and extension.

**Figure 2:**
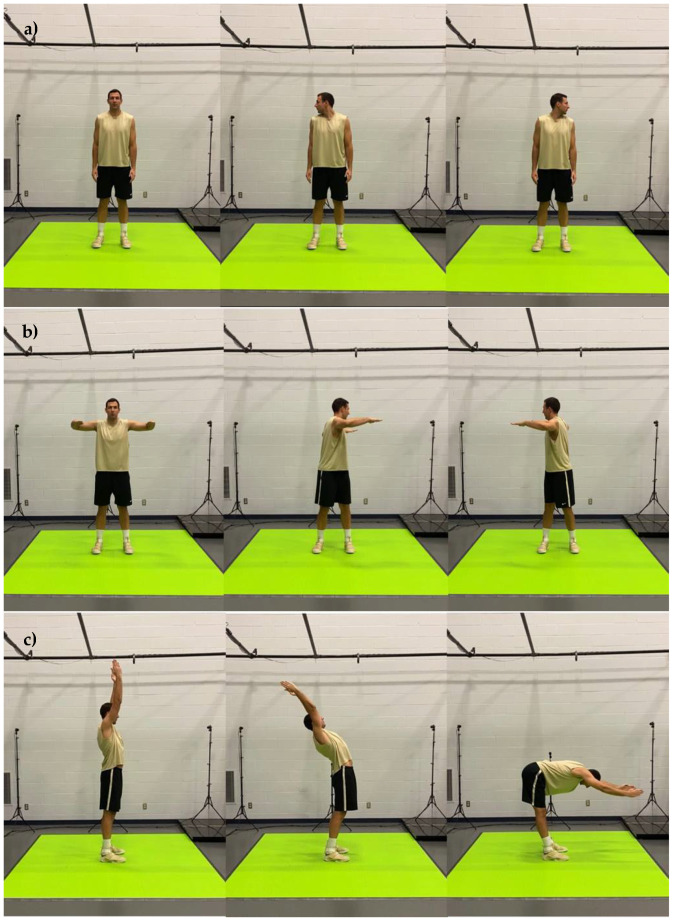
Graphical representation of body movements incorporated in motion health screening protocol; (**a**) cervical rotation; (**b**) trunk rotation; (**c**) trunk extension and flexion.

**Figure 3:**
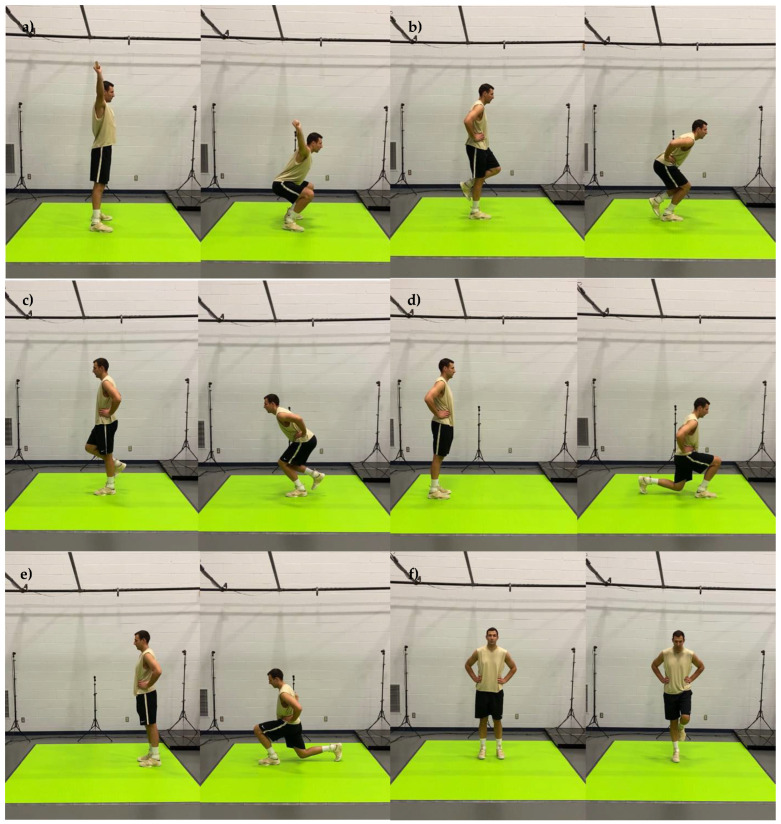
Graphical representation of body movements incorporated in motion health screening protocol; (**a**) bilateral squat, (**b**) right leg squat, (**c**) left leg squat, (**d**) right leg lunge, (**e**) left leg lunge, (**f**) right leg balance.

**Figure 4:**
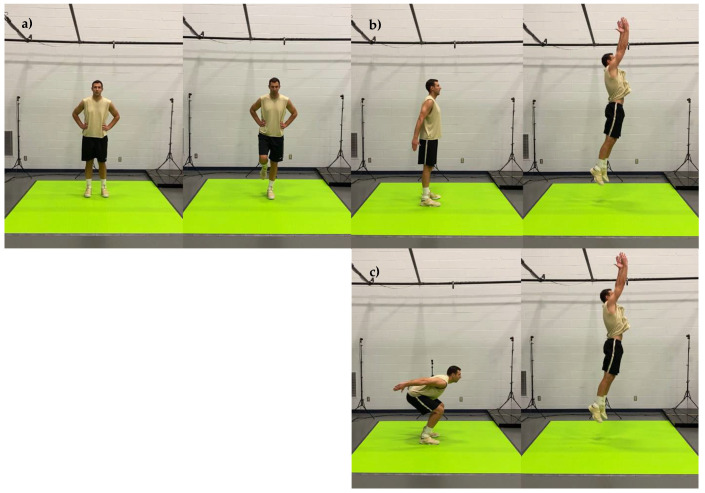
Graphical representation of body movements incorporated in motion health screening protocol; (**a**) left leg balance, (**b**) bilateral standing vertical jump, (**c**) concentric jump.

**Figure 5:**
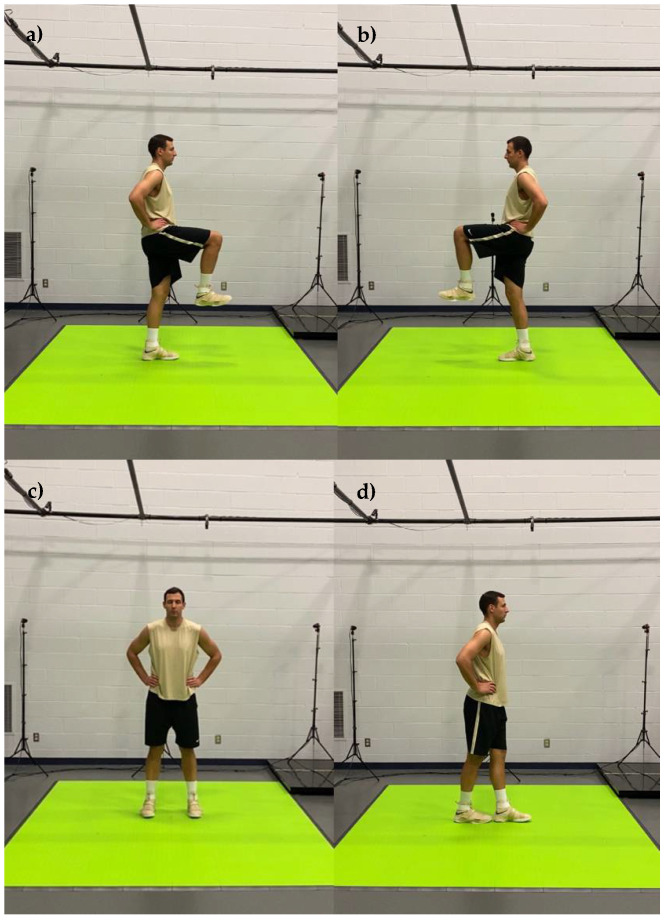
Graphical representation of body movements incorporated in motion health screening protocol; (**a**) left stork stance, (**b**) right stork stance, (**c**) standing double leg balance, (**d**) tandem stance balance.

The motion capture screenings consisted of 20 body movements performed in sequential order. The title and a brief description of each movement are presented in Table 1 and the detailed graphical representation in Figure 1, Figure 2, Figure 3, Figure 4 and Figure 5. The order in which motions were performed was identical across all participants. A three-dimensional markerless motion capture system (DARI Motion, Overland Park, KS, USA), composed of eight high-definition cameras recording at 60 fps, was used to obtain the biomechanical parameters (i.e., kinetics and kinematics) of each motion. Prior to each testing session, system calibration was performed following manufacturer-based recommendations. The data collection and analysis resembled the methodology previously used by Cabarkapa et al. [15]. The cameras were positioned at different orientations to surround and cover the testing/screening area. For the purpose of consistency, during each of the six separate motion capture screenings, a trained research team member, facing the participant and standing outside of the camera capture range, was present to demonstrate each individual movement. Following the demonstration, the participant received the command “one, two, three, begin”. At the command “begin”, the second member of the research staff began motion capture, and the participant started the specific movement. After the completion of the movement, the research team member gave the command “done”, as a signal for the second member of the research team to stop the motion-related recording. 

After the completion of the health motion screening protocol, a plethora of kinetic and kinematic parameters, obtained during 20 body movements incorporated into the screening protocol, were automatically processed (~30 s) by DARI Motion-defined algorithms to derive the five unique scoring scales/variables: explosiveness, functionality, dysfunction, quality, and readiness. The explosiveness scale represents jump-related performance parameters (e.g., jump heights). The functionality scale is an aggregate of all squat-related motions (e.g., squat depth). The dysfunction scale is a summary of motion and balance-related asymmetries/alignment (e.g., lower-limb asymmetries, knee valgus). The readiness scale is a cumulative score based on the overall performance capture analysis (i.e., readiness = explosiveness + functionality − dysfunction). Lastly, the quality scale assesses the movement patterns across all movements completed throughout the screening protocol and takes into account stress-related captures (e.g., high unilateral forces) and compensation patterns (e.g., overuse or limited usage due to a history of injury). The quality scale is expressed as a percentage (0–100% range), while the rest of the scales are represented in arbitrary units (a.u.).

### 2.3. Statistical Analysis 

After the visual data inspection, two data points (i.e., one functionality and one readiness score) were identified as obvious outliers (i.e., greater than four standard deviations from the mean) and were replaced by an average value of the scores obtained across the five remaining screening sessions. Descriptive statistics, means and standard deviations ($\bar{x}$ ± SD), were calculated for each dependent variable. The Shapiro–Wilk test and Q–Q plots corroborated that the assumption of normality was not violated. Repeated measures analysis of variance (ANOVA) with Bonferroni post-hoc adjustments was used to examine statistically significant differences for each dependent variable across six testing sessions. Mauchly’s Test of Sphericity was used to test whether or not the assumption of sphericity was met. If violated, Greenhouse–Geisser adjustment for lack of sphericity was used. In addition, an intraclass correlation coefficient (ICC) with 95% confidence intervals (95% CI) was used to examine the measurement agreement for each dependent variable across six testing sessions. Statistical significance was set a priori to *p* < 0.05. All statistical analyses were completed with SPSS (Version 26.0; IBM Corp., Armonk, NY, USA) and Microsoft Excel (Microsoft Corp., Redmond, WA, USA).

## 3. Results

**Figure 6:**
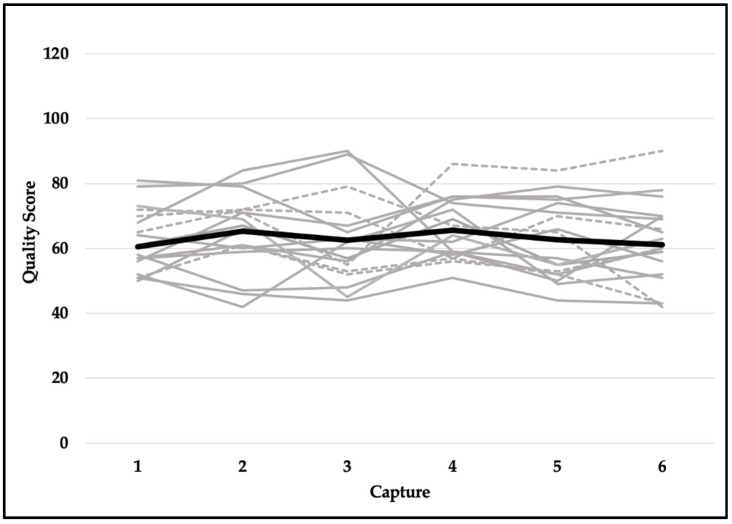
Quality scores across six capture time points. Gray lines represent values for each subject (solid—female; dashed—male) and the black bolded line represents the overall mean.

**Figure 7:**
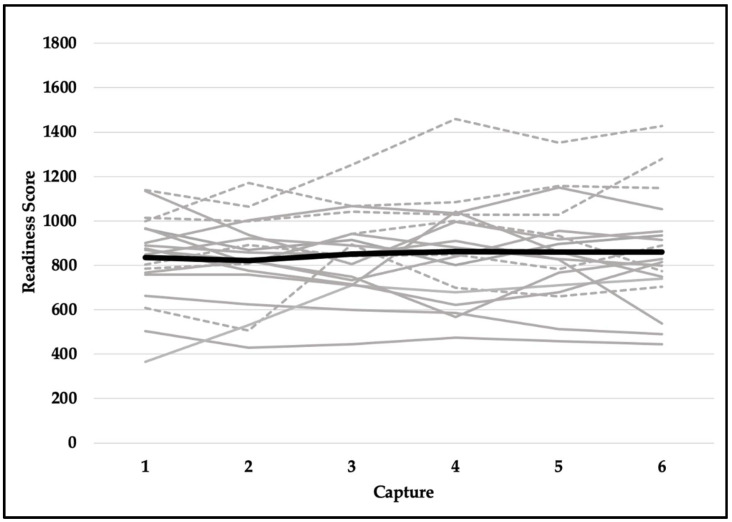
Readiness scores across six capture time points. Gray lines represent values for each subject (solid—female; dashed—male) and the black bolded line represents the overall mean.

**Figure 8:**
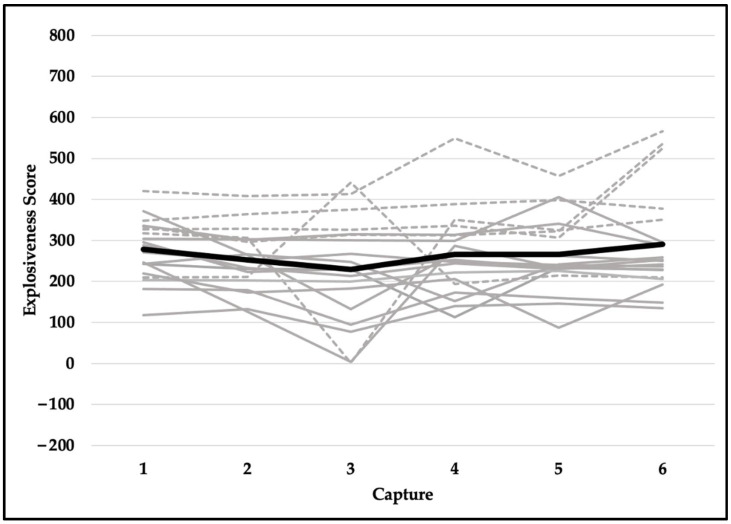
Explosiveness scores across six capture time points. Gray lines represent values for each subject (solid—female; dashed—male) and the black bolded line represents the overall mean.

**Figure 9:**
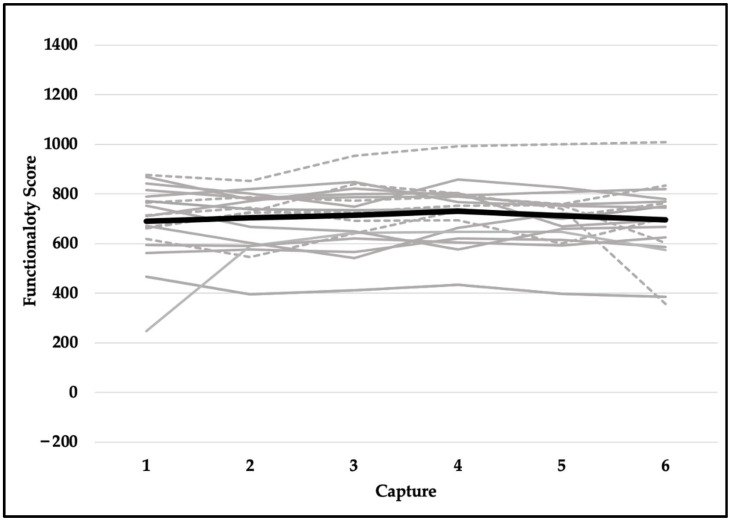
Functionality scores across six capture time points. Gray lines represent values for each subject (solid—female; dashed—male) and the black bolded line represents the overall mean.

**Figure 10:**
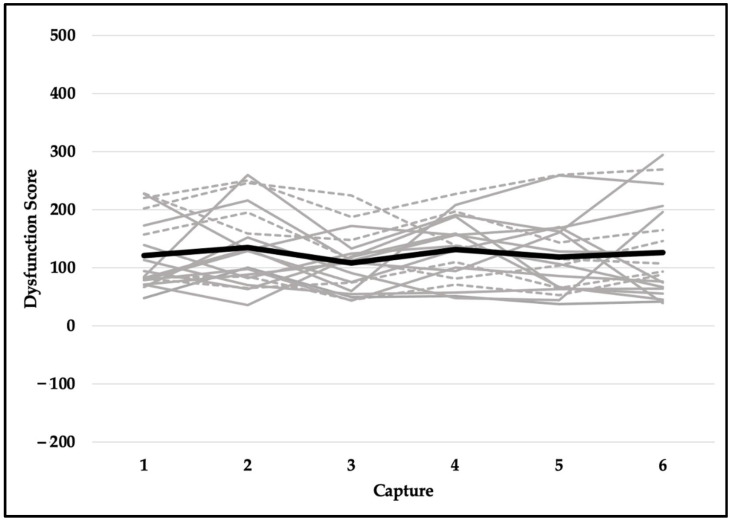
Dysfunction scores across six capture time points. Gray lines represent values for each subject (solid—female; dashed—male) and the black bolded line represents the overall mean.

Quality scores met the assumption of sphericity (*χ^2^*_(14)_ = 18.471, *p* = 0.191) and were not significantly different across six testing sessions (F_(5,90)_ = 1.037, *p* = 0.401). Despite violating the assumption of sphericity (*χ^2^*_(14)_ = 23.937, *p* = 0.049), the difference in readiness scores was not statistically significant (F_(5,90)_ = 0.563, *p* = 0.667). Explosiveness scores did not meet the assumption of sphericity (*χ^2^*_(14)_ = 42.022, *p* < 0.001) and did not reach the level of statistical significance (F_(5,90)_ = 2.222, *p* = 0.109). Although functionality scores violated the assumption of sphericity (*χ^2^*_(14)_ = 39.543, *p* < 0.001), they were not significantly different across six testing sessions (F_(5,90)_ = 0.739, *p* = 0.530). Dysfunction scores did not meet the assumption of sphericity (*χ^2^*_(14)_ = 33.566, *p* = 0.003) and were not statistically significant (F_(5,90)_ = 0.758, *p* = 0.529). In addition, reliability for readiness scores was excellent (ICC, 95% CI; 0.957, 0.914–0.980), good-to-excellent for functionality (0.905, 0.821–0.959) and explosiveness (0.906, 0.822–0.959) scores, and moderate-to-excellent for dysfunction (0.829, 0.675–0.925) and quality (0.808, 0.635–0.915) scores. See Table 2 for means and standard deviations and Figure 6, Figure 7, Figure 8, Figure 9 and Figure 10 for a detailed graphical representation of the results of each dependent variable across six testing time points.

## 4. Discussion

The findings of the present study indicate no statistically significant differences in algorithm-based motion health screening scores derived from a three-dimensional markerless motion capture system across six testing sessions. Moreover, excellent measurement reliability was found for readiness scores, good-to-excellent for functionality and explosiveness scores, and moderate-to-excellent for dysfunction and quality scores [16]. 

Hando et al. [17] used an identical markerless motion capture system (i.e., DARI Motion) to screen for musculoskeletal injury risk within a large cohort of military trainees, as well as to examine the test-retest reliability of the system. Alongside poor-to-moderate test-retest reliability scores, it has been found that the assessment scores obtained from the markerless motion capture system were not able to discriminate between individuals who were and were not likely to suffer musculoskeletal injury [17]. These results are contradictory to the findings of the present study that found moderate-to-excellent, good-to-excellent, and excellent measurement reliability. The observed discrepancy may be attributed to a number of body movements incorporated into the motion screening protocol and the descriptions/type of instructions provided to each participant. Hando et al. [17] incorporated eight body movements into their motion screening protocol (i.e., shoulder internal/external rotation, shoulder flexion/extension, overhead squat, unilateral squat right/left, vertical jump, and unilateral vertical jump right/left), while the protocol performed in the present study was composed of 20 body movements (Table 1), thereby allowing for a more thorough assessment of kinetic and kinematic characteristics used to derive algorithm-based performance scores. Moreover, although the participants should not be coached on how to properly perform each body movement for the purpose of accurate performance assessment, they should be provided with elementary instructions that are easily understandable by a broad spectrum of the population. Knowing the starting position and in which anatomical plane of motion the movement is performed is necessary for the proper assessment of the biomechanical characteristics of each motion (e.g., start with arms in a T-position, raise arms overhead, and lower hands to the sides). This may also minimize the influence of the learning effect, resulting from performing the same movement multiple times. Moreover, it is of critical importance that the instructions on how to perform a specific movement remain consistent across all participants and testing sessions. 

In a recently published study, Bird et al. [1] examined the relationship between movement strategies during countermovement vertical jumps and musculoskeletal injury risk among marine corps officer candidates. Although the screening procedure included force plate testing (e.g., force, power, rate of force development), kinematic variables of interest (e.g., hip, knee, and ankle flexion, and dynamic valgus) obtained from an identical motion capture system to the one used in the present study (i.e., DARI Motion) were capable of successfully differentiating between individuals with a low, moderate, and high risk of musculoskeletal injury [1]. On the other hand, when used in a clinical setting, it has been found that algorithm-derived variables obtained from the same markerless motion capture system (e.g., mobility, alignment, readiness) were capable of tracking gradual recovery progress throughout a 3–6-month period, post anterior cruciate ligament reconstruction [2]. The authors also alluded that utilization of this innovative motion-tracking technology can aid with an objective decision-making process and minimize subjective review of a patient’s progress, ultimately decreasing the likelihood of reinjury occurrence [2]. In addition, although further research is warranted on this topic, Martinez et al. [18] have found that kinematic data obtained from a markerless motion capture system (e.g., shoulder flexion, shoulder rotation) could serve as a supplemental screening modality, aiding in the process of discriminating between patients with Parkinson’s disease and healthy controls. Overall, these findings further support the application of markerless motion capture technology for the assessment of various types of biomechanical parameters of human motion in the applied setting (e.g., military and clinical settings). 

Another important factor that should be considered when examining the measurement reliability of any system is the distinction between biological and technological variability. Although the usage of markerless motion capture technology eliminates the measurement error related to marker application/reapplication and skin marker movement, the inherent biological variability in human locomotion is inevitable [11]. Yet, no statistically significant changes in algorithm-derived motion capture scores were observed in the present study, suggesting that the instructions for performing each body motion were consistent and/or the system was resilient to this source of variability. On the other hand, despite using an identical motion capture system throughout all testing procedures, the technological variability of this markerless motion capture system was not examined in the present study. This presents a limitation of the present investigation and provides one of the directions for future research (e.g., variability in motion health screening scores assessed by two identical systems, recording the same motion at the same time). Moreover, although the present study examined the reliability of algorithm-based motion capture scores (i.e., quality, readiness, explosiveness, functionality, and dysfunction), future research should examine the repeatability of raw kinetic and kinematic variables obtained from this type of markerless motion capture system (e.g., shoulder flexion/extension, bilateral squat depth, trunk rotation), as well as its ability to accurately capture minimal detectable and/or clinically relevant changes in performance. 

## 5. Conclusions

The findings of the present study indicate no statistically significant differences in algorithm-based motion health screening scores, derived from a three-dimensional markerless motion capture system across six testing sessions. Excellent measurement reliability was found for readiness scores, good-to-excellent for functionality and explosiveness scores, and moderate-to-excellent for dysfunction and quality scores. Although further work is warranted to more thoroughly determine biological and technological variability associated with this assessment modality, these findings support the application of markerless motion capture technology for the assessment of various types of biomechanical parameters of human motion in the applied setting.

## Figures and Tables

**Table 1:** List and description of body movements incorporated in motion health screening protocol.

Specific Body Movement	Description of Movement
Shoulder abduction and adduction	Start with arms out in a T-position, raise arms overhead and lower hands to sides.
Shoulder horizontal abduction	Start with arms straight-out in front of body, separating arms, reach back behind body and return to the starting position.
Shoulder internal and external rotation	Start with arms in a goalpost position, holding this position, rotate arms upward and back, followed by rotating arms forward and down.
Shoulder flexion and extension	Start with arms to side and with palms facing inward, raise arms upward as far as possible and then back as far as possible.
Cervical rotation	Start with the head facing forward and rotate the head as far to the right as possible, then as far to the left as possible.
Trunk rotation	Start with arms upward and in a goalpost position, then rotate as far as possible to the left and then as far as possible to the right, keeping hips in a forward position.
Trunk extension and flexion	Start with arms overhead and lean back as far as possible, then return to start position and bend forward as far as possible (trying to touch toes), keeping legs as straight as possible.
Bilateral squat	Start with feet forward and shoulder distance apart, and while holding a light bar directly above head, lower body downward as far as possible.
Right leg squat	Start by raising left foot off the ground and while balancing on the right leg, lower body down as far as possible on the standing leg and return to the starting position.
Left leg squat	Start by raising right foot off the ground and while balancing on the left leg, lower body down as far as possible on the standing leg and return to the starting position.
Right leg lunge	Start with body and feet in a forward position, then take a big step forward with right foot only and lower body toward the ground. Return to the starting position.
Left leg lunge	Start with body and feet in a forward position, then take a big step forward with left foot only and lower body toward the ground. Return to the starting position.
Right leg balance	Start with the body and feet in a forward position, then raise the left slightly off the ground. Standing on the right leg, balance body for 30 s. Hopping on one leg is allowed as long as the left foot does not touch the ground.
Left leg balance	Start with body and feet in a forward position, then raise the right leg slightly off the ground. Standing on the left leg, balance body for 30 s. Hopping on one leg is allowed as long as the right foot does not touch the ground.
Bilateral standing vertical jump	Start with feet forward, legs straight and arms extended backwards as far as possible, then jump as high as possible off both legs.
Concentric jump	Start with feet forward, legs bent to a near 90-degree angle and arms extended backwards as far as possible, then jump as high as possible off both legs.
Right stork stance	Start with body and feet in a forward position, then raise the left leg upward to a near 90-degree angle. Close eyes and standing on the right leg, balance body for 20 s. Hopping on one leg is allowed as long as left foot does not touch the ground.
Left stork stance	Start with body and feet in a forward position, then raise the right leg upward to a near 90-degree angle. Close eyes and standing on the left leg, balance body for 20 s. Hopping on one leg is allowed as long as right foot does not touch the ground.
Standing double leg balance	Start with body and feet in a forward position. With feet together, close eyes and balance body for 20 s.
Tandem stance balance	Start with body in a forward position and place one foot directly in front of the other foot in a forward position. Close eyes and balance body for 20 s.

**Table 2:** Descriptive statistics ($\bar{x}$ ± SD) for each dependent variable.

**Dependent Variable**	**Capture 1**	**Capture 2**	**Capture 3**
Quality	60.6 ± 12.1	65.4 ± 11.5	62.5 ± 13.0
Readiness	834.6 ± 198.8	821.1 ± 191.6	850.9 ± 186.7
Explosiveness	277.7 ± 73.7	252.7 ± 74.9	229.7 ± 126.3
Functionality	690.3 ± 151.3	702.9 ± 123.3	713.3 ± 126.9
Dysfunction	121.0 ± 60.9	134.5 ± 69.3	107.9 ± 50.6
**Dependent Variable**	**Capture 4**	**Capture 5**	**Capture 6**
Quality	65.7 ± 9.2	62.7 ± 11.8	61.2 ± 12.9
Readiness	863.6 ± 232.3	859.5 ± 217.9	860.4 ± 249.1
Explosiveness	265.7 ± 101.6	266.4 ± 93.0	290.8 ± 126.2
Functionality	729.4 ± 121.5	711.8 ± 122.4	695.3 ± 155.5
Dysfunction	131.5 ± 55.8	118.7 ± 66.1	126.1 ± 80.8

## Data Availability

The data presented in this study are available on request from the corresponding author.
